# Effective *W*-state fusion strategies for electronic and photonic qubits via the quantum-dot-microcavity coupled system

**DOI:** 10.1038/srep12790

**Published:** 2015-08-05

**Authors:** Xue Han, Shi Hu, Qi Guo, Hong-Fu Wang, Ai-Dong Zhu, Shou Zhang

**Affiliations:** 1 Department of Physics, College of Science, Yanbian University, Yanji, Jilin 133002, China; 2 Department of Physics, Harbin Institute of Technology, Harbin, Heilongjiang 150001, China

## Abstract

We propose effective fusion schemes for stationary electronic *W* state and flying photonic *W* state, respectively, by using the quantum-dot-microcavity coupled system. The present schemes can fuse a *n*-qubit *W* state and a *m*-qubit *W* state to a (*m* + *n* − 1)-qubit *W* state, that is, these schemes can be used to not only create large *W* state with small ones, but also to prepare 3-qubit *W* states with Bell states. The schemes are based on the optical selection rules and the transmission and reflection rules of the cavity and can be achieved with high probability. We evaluate the effect of experimental imperfections and the feasibility of the schemes, which shows that the present schemes can be realized with high fidelity in both the weak coupling and the strong coupling regimes. These schemes may be meaningful for the large-scale solid-state-based quantum computation and the photon-qubit-based quantum communication.

Entanglement is a unique phenomenon in quantum mechanics, and it is an important quantum resource in the quantum information field. Especially, quantum entanglement plays a vital role in quantum communication and quantum information processing (QIP), such as quantum computation^1^, quantum teleportation^2^, quantum key distribution (QKD)^3^, and so on. It is known to all that bipartite entanglement is different from multipartite entanglement. Among the multipartite entangled states, *W* state, GHZ state and cluster state form inequivalent classes and they can’t be transformed into each other by local operations and classical communication. *W* state is a special kind of entangled state in the multipartite system. Compared with the GHZ state, *W* state is highly robust against the qubits loss. Hence, *W* state has recently attracted considerable attention in the field of quantum computing and information science^4^,^5^,^6^,^7^. For example, *W* state has been used for the optimal universal quantum cloning machine^8^ and has also been proposed as a resource for QKD^9^. The more the number of particles forming an entangled state is, the more complex the entanglement structures are. Therefore the creation of multipartite entangled states has been paid much attention.

In the last decade, expansion and fusion operations have been proposed and demonstrated as efficient ways to prepare large scale multipartite entangled states. One can get a larger entangled state from two or more qubits entangled states by fusion operation on the condition that the access is granted only to one qubit of each of the states entering the fusion operation. Nowadays, much attention has been paid to the preparation of large scale multipartite entangled states by fusion operation. Currently, many expansion and fusion proposals of multipartite entangled states have been put forward, such as using cluster states with smaller-scale qubits to prepare larger cluster states^10^, the creation of large-scale GHZ states^11^ and *W* states^12^,^13^,^14^,^15^,^16^,^17^,^18^,^19^,^20^. Among those schemes, in 2011 Ozdemir *et al.* first used a simple optical fusion gate to get a *W* state *W*_*n*+*m*−2_ from *W*_*n*_ and *W*_*m*_ (*n*,*m* ≥ 3 and *W*_*x*_ denotes a *x*-qubit *W* state)^16^. In the following years, they put forward several *W* states fusion schemes with the help of complex quantum gate sets. However, multi-qubit controlled gates are great challenges for linear optical quantum computation^3^. Recently, we proposed a *W*-state fusion scheme without multi-qubit logical gates with the help of weak cross-Kerr nonlinearities^20^.

Here, we respectively propose fusion schemes of electron-spin and polarized-photon *W* states with the help of quantum-dot(QD)-microcavity coupled system. Compared with the previous work^20^, the present schemes not only propose the fusion scheme of polarized-photon *W* state, but also propose the fusion scheme of electron-spin *W* state, which can be used to solid-state-based quantum information processing. These schemes may be meaningful for the large-scale solid-state-based quantum computation and the photon-qubit-based quantum communication. In these schemes, only one particle of each multipartite entangled states is sent into the fusion device. After a series of operations, only one of the two particles is detected and the other is returned back to the state to prepare a (*m* + *n* − 1)-qubit *W* state. Due to the developments in semiconductor nanoelectronics technology, QD-microcavity coupled system has been widely studied as a promising physical system for solid-state-based quantum information processing. So far, many efforts have been made such as fast initialization of the spin state of a single electron^21^, nondestructive measurement^22^, and fast optical control and coherent manipulation of a QD spin^23^,^24^. In recent years, a series of quantum information processing schemes based on QD-microcavity coupled system have been proposed^25^,^26^,^27^,^28^,^29^,^30^,^31^,^32^,^33^,^34^,^35^,^36^,^37^. All these works indicated that QD-microcavity coupled system is a good promising candidate and can be used for the storage and manipulation of quantum information. Therefore, it’s meaningful to study the large-scale entanglement creation and fusion in the QD-microcavity system. Although the universal quantum gates can generate arbitrary entangled states, here we directly fuse the large-scale *W* states without any universal quantum gates which reduce the difficulty of experiment and can be achieved under the current experiment technology.

## Results

### Electronic *W*-state Fusion based on QD-microcavity coupled system

**Figure 1:**
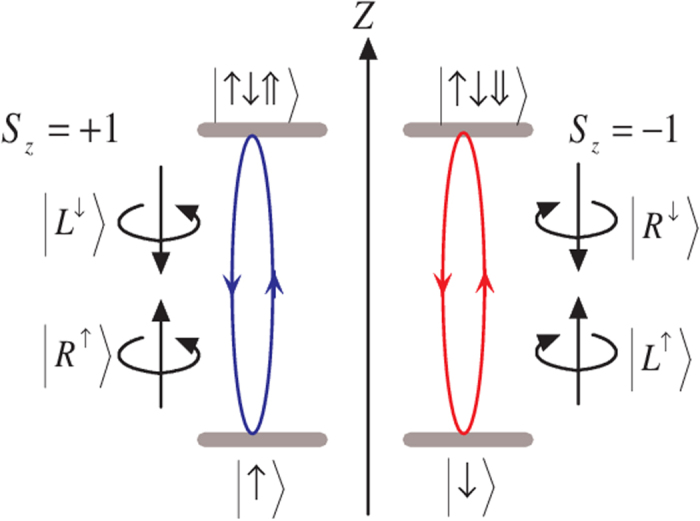
Relevant energy levels and optical selection rules for the optical transition of negatively charged exciton *X*^−^ in GaAs/InAs quantum dots embedded in an optical microcavity. The superscript arrows of the photon states indicates their propagation direction along or against the *z* axis.

Here, we consider a singly charged GaAs/InAs QD, which has four relevant electronic levels, 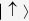, 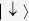, 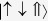, and 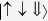, as shown in Fig. 1, being embedded in a double-sided optical microcavity with both the top and bottom mirrors partially reflective. The negatively charged exciton (*X*^−^), produced by the optical excitation of the system, contains two electrons bound in one hole. 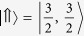 and 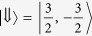 represent heavy hole states with spin 3/2 and 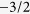 components. The total spin of the two electrons in an exciton is zero, which prevents the interaction between the electron-spin and the heavy hole spin. The quantization axis for angular momentum is the *z* axis because the quantum dot confinement potential is much tighter in the *z*(growth) direction than in the transversal direction due to the quantum dot geometry. According to this feature, it has two optical transitions between the electron state and the exciton state by involving the photon whose spin is *s*_*z*_ = +1 (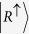 or 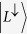) or *s*_*z*_ = −1 (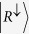 or 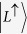). Based on the optical selection rules and the transmission and reflection rules of the cavity for an incident circular polarization photon, the interactions between photons and electrons in the QD-microcavity coupled system can be described as follows^26^,^27^:


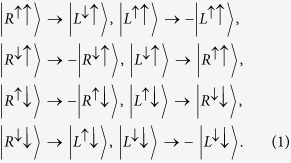


where 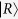 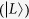 denotes the right(left)-circularly polarized photon state. The superscript up arrow (down arrow) denotes the propagating direction of polarized photon along (against) the *z* axis.

**Figure 2:**
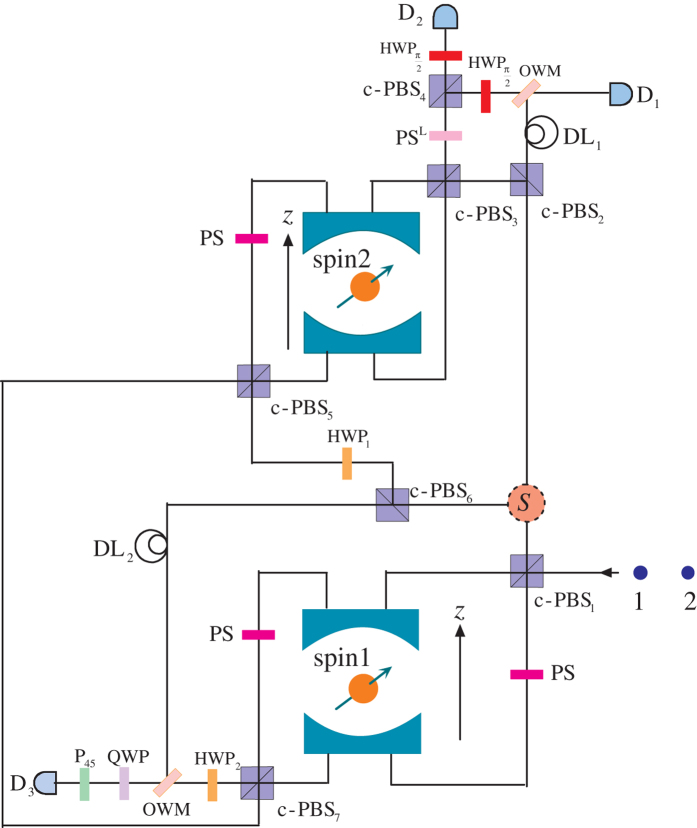
Schematic of electronic *W*-state. Spin 1 and spin 2 denote two QD spins coupled with two optical microcavities, respectively. c-PBS_*i*_ (i = 1, 2, 3…): polarizing beam splitter in the circular basis. HWP_*i*_ is half-wave plate, and 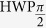 implements the transformation 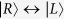. QWP: quarter-wave plate. S: optical switch. PS: *π*-phase shifter. The PS^*L*^ introduces a *π* phase for 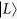 and does not affect 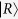. OWM: one-way mirror transmits photons from one side, and reflects photons from the other side without remodulating. P_45_ is a 45° polarizer projecting the polarization to 
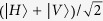
. DL_*i*_: the time-delay device for matching pathes length of the different components of the same photon. D_*i*_: conventional photon detectors.

Now, we introduce how to implement a (*m* + *n* − 1) qubits electronic *W*-state fusion scheme from *m* qubits *W* state and *n* qubits *W* state based on QD-microcavity coupled system. The schematic is depicted in Fig. 2. We consider two spatially separated parties, Alice and Bob, who dominate two electronic entangled *W* states, 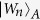 and 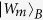, respectively, and decide to fuse their entangled states 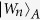 and 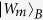 to get a larger *W* state. The electronic *W* states of Alice and Bob can be denoted as^14^


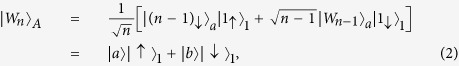



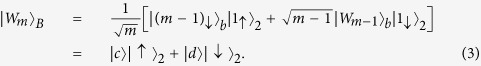


In this notation, a tripartite *W* state is written as 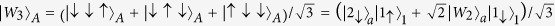 with 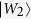 corresponding to the EPR pair 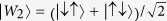. For simplicity, here we have substituted 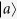 for 
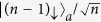
, 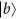 for 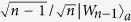, 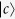 for 
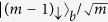
, and 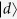 for 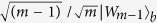. With the help of two ancillary photons in right-circular polarization state 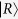, we can fuse Alice’s and Bob’s electronic *W* states to a larger *W* state 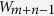. The initial state of the whole system is


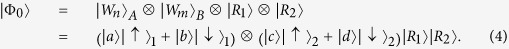


In the fusion process, only the electron spin 1 and 2 interaction with photons, the remaining electrons in modes *a* (*b*) are kept constant at Alice’s (Bob’s) side. Namely, only 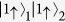, 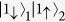, 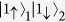, and 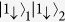 interact with the photons, whose probabilities are 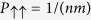, 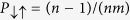, 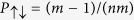, and 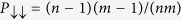, respectively.

Firstly, photon 1 passes through the c-PBS_1_, which is polarizing beam splitter in the circular basis transmiting the right-circularly-polarized photon 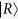 and reflecting the left-circularly-polarized photon 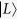. That is, the components 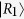 and 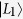 enter the cavity 1 from the top and the bottom, respectively. PS is a phase shifter that contributes a *π* phase shift to the photon passing through it (i.e., 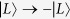 and 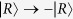). After the interaction with cavity 1, the components 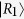 and 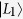 mix at c-PBS_1_ again. The above operations 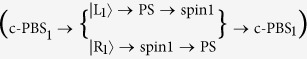 transform 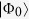 into


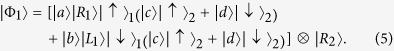


Then, photon 1 gets to the optical switch (S) and goes toward the c-PBS_2_. For the component 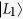, it passes through c-PBS_3_ and enters cavity 2 to interact with spin 2. After that, it passes through the PS^*L*^, c-PBS_4_ and 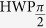 in turn. Here, the PS^*L*^ implements transformations 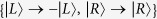 and 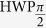 is a half-wave plate oriented at 45° and realizes the transformation 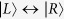. That means the sate including component 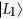 after these operations (c-PBS_2_ → c-PBS_3_ → spin2 → PS^*L*^ → c-PBS_4_ → 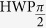) becomes 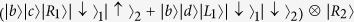. While the component 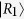 transmits from c-PBS_2_ and does not interact with the cavity. It passes c-PBS_2_ → DL_1_ → OWM in turn. DL is the time-delay device for matching path lengths of the two components. OWM is one-way mirror transmits photons from one side, and reflects photons from the other side without remodulating^38^,^39^. Hence, before the detectors click, the state will be


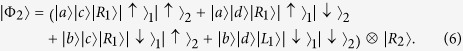


It is obvious that when the detector D_2_ detects photon, the state is


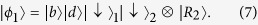


In this case, we will obtain two separate *W* states with a smaller number of qubits, 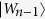 and 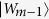, which can be recycled using the same fusion mechanism. However, when the detector D_1_ detects photon, the state of the system is changed as


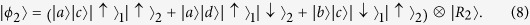



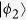
 is used to continue the fusion process. Now, let the photon 2 pass through c-PBS_1_ and interact with the spin 1. After passing though c-PBS_1_ again, we obtain


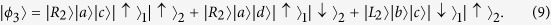


Then, the photon 2 exits from the optical switch and goes towards c-PBS_6_. The component 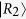 transmits c-PBS_6_ and does not interact with the spin 2 and spin 1. However, the component 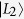 passes through HWP_1_ which realizes 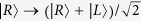 and 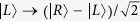, and then enters cavity 2 via c-PBS_5_ to interact with the spin 2. It is worth noting that before and after the photon 2 interacts with the electron spin 2, a Hadamard operation (H_*e*_) on the electron spin 2 is performed by a *π*/2 microwave pulse or an optical pulse^23^,^40^, i.e. 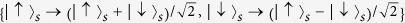. With operations 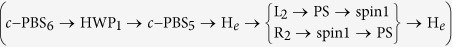, the photon-electron state can evolve as


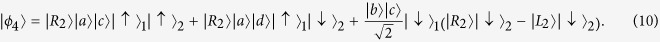


Whereafter, the photon passes c-PBS_7_ and PS, successively. Before and after interacting with the spin 1, we performs a Hadamard gate on electron spin 1. Then the photon 2 passes through the HWP_2_. With these operations (c-PBS_7_ → H_*e*_ → spin 1 → H_*e*_ → HWP_2_), we obtain


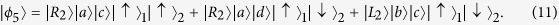


Due to the DL, the photon components exit from c-PBS_7_ and c-PBS_6_ and arrive the OWM at the same time. QWP is a quarter-wave plate which implements transformations 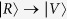 and 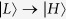. P_45_ is a 45° polarizer^41^ projecting the polarization to 
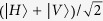
. The photon passing through the OWM, QWP and P_45_ in turn. As long as the photon detector D_3_ clicks, the resulting state of the total spin-photon system becomes as:


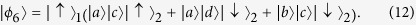


It is clearly that electron spin 1 is disentangled with other electron spins. Therefore the state of the remaining electron spins is given by


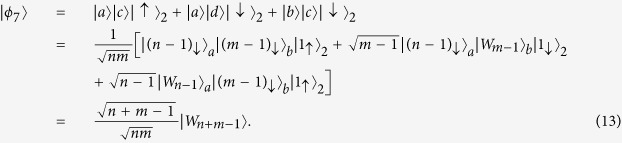


Obviously, 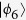 is a *W* state with *n* + *m* − 1 electrons (i.e., 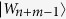), and the probability obtaining the *W* state is (*n* + *m* − 1)/*nm*. Here we have used 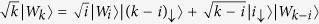.

### Photonic *W*-state Fusion based on QD-microcavity coupled system

**Figure 3:**
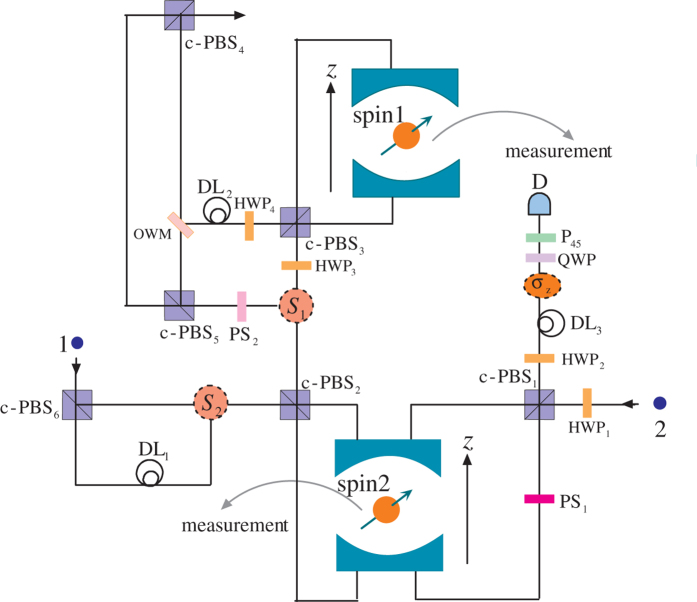
Schematic of photonic *W*-state fusion. All the optical elements are the same as Fig. 2.

In this section, we will introduce the fusion protocol of the photonic *W*-state in detail. The schematic is depicted in Fig. 3. We use the similar notation 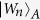 and 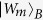 as electronic *W* states in the above section,


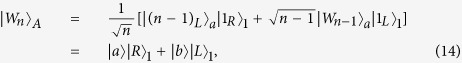



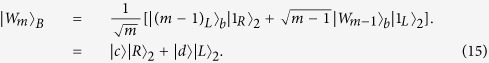


Distinctly, a tripartite *W* state is written as 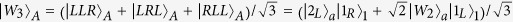 with 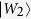 corresponding to the EPR pair 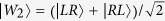. For simplicity, here we have substituted 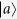 for 
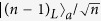
, 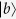 for 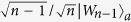, 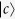 for 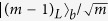, and 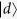 for 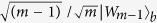. Our scheme is based on the QD double-side optical microcavity, the electron spin 1 (electron spin 2) is initially in the state 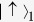 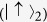. Therefore the initial state can be written as:


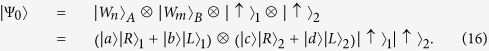


In our fusion scheme, only the photons in mode 1 and 2 are sent to the fusion device and the remaining photons in modes *a* (*b*) are kept constant at Alice’s (Bob’s) side. In other words, only 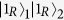, 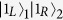, 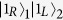, and 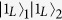 are sent to the fusion mechanism with the probabilities *P*_*RR*_ = 1/(*nm*), *P*_*LR*_ = (*n* − 1)/(*nm*), *P*_*RL*_ = (*m* − 1)/(*nm*), and *P*_*LL*_ = (*n* − 1)(*m* − 1)/(*nm*), respectively.

Before and after the photon 2 passes through the second optical microcavity, Hadamard operations H_*p*_ and H_*e*_ are performed on photon 2 and electron 2, respectively. PS_1_ realizes the transformations 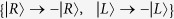. After these operations 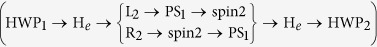, the state of the photon-electron system is given by


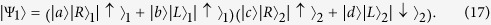


Now, the photon 1 passes through the c-PBS_6_, the component 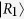 does not interact with the spin 2 and spin 1 while the component 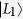 passes through the optical switch S_2_ and the c-PBS_2_. Then it enters cavity 2 and interacts with the spin 2. These operations (c-PBS_6_ → S_2_ → c-PBS_2_ → spin 2 → c-PBS_2_) make 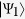 become


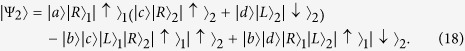


Next, the photon 1 goes through the optical switch S_1_, HWP_3_, c-PBS_3_, one by one. Before and after the photon 1 interacts with the electron spin 1, a Hadamard operation H_*e*_ is performed on the electron spin 1 by using a *π*/2 microwave pulse or an optical pulse. Then photon 1 passes HWP_4_. After these operations (S_1_ → HWP_3_ → H_*e*_ → c-PBS_3_ → spin 1 → H_*e*_ → c-PBS_3_ → HWP_4_), the state becomes


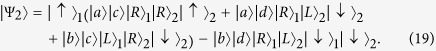


Here, a measurement is performed on the electron spin 1 which has two possible results as below


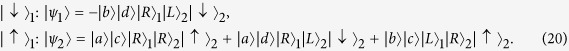


For the result 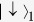, we will get two separate *W* states with a smaller number of qubits, 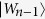 and 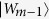, which can be recycled by the same fusion mechanism. For the other situation we acquire 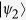, it will be used to continue the fusion process. At this time, the component 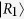 in the *DL*_1_ passes through the optical switch S_2_, c-PBS_2_ and enters the spin 2. When the photon leaves away from the cavity 2, it passes through the switch S_1_ and interacts with PS_2_ which implements the transformations {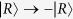, 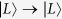}. After these operations (c-PBS_2_ → spin 2 → c-PBS_2_ → S_1_ → PS_2_ → c-PBS_5_ → OWM), the photon-electron state evolves as


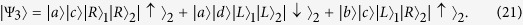


Here, the DL_2_ makes the components of photon 1 which exit from the HWP_4_ and PBS_5_, respectively, to arrive the OWM at the same time. Now, all the components of the photon 1 are combined at c-PBS_4_.

Then we perform a Hadamard gate on electron spin 2 and measure it in basis 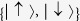. If the measurement result is 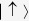, the state of the remaining system is


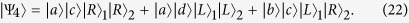


Now, the photon 2 derectly passes through QWP and P_45_ in turn. When the photon detector D clicks, the resulting state of the remaining photons becomes as below:


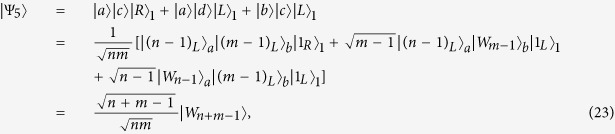


which is a *W* state with *n* + *m* − 1 photons, i.e. 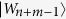, where we have used 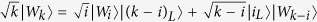. If the electron 2 is in 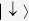, we have


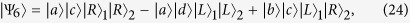


The *σ*_*z*_ operation on the photon 2 is needed before it passes through QWP, and the state of photons will become the same as Eq. (22). With the same operation above, we can obtain a (*n* + *m* − 1)-qubit *W* state in Eq. (23) with the probability (*n* + *m* − 1)/*nm*. So far, we have completed the *W*-state fusion schemes for electronic and photonic *W* state, respectively, based on the quantum-dot-microcavity coupled system.

## Discussion

In this section, we will briefly analyze and discuss the feasibility and the success probability of the proposed schemes. When the side leakage and cavity loss are taken into account, the reflection and transmission coefficients of the coupled and the uncoupled cavities are generally different in a realistic *X*^*−*^-cavity system. The reflection and transmission coefficients of a double-sided optical microcavity for weak excitation limit can be described by^26^,^27^


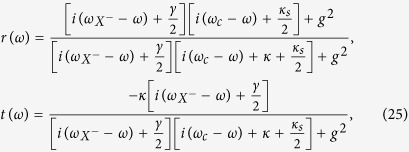


here *g* is the coupling strength, *κ*, *κ*_*s*_, and *γ* are the cavity field decay rate, leaky rate, and *X*^−^ dipole decay rate, respectively. *ω*, *ω*_*c*_, and 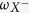 are the frequencies of the input photon, cavity mode, and the spin-dependent optical transition, respectively. By setting 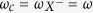, the reflection and transmission coefficients of the coupled cavity is given by


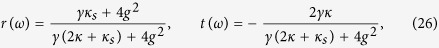


and uncoupled cavity (*g* = 0) is


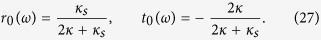


Therefore, in a realistic spin-QD-double-side-cavity unit, the rules of the optical transitions can be described as


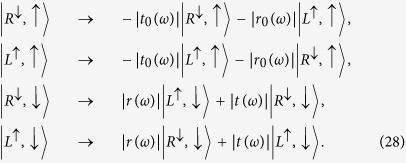


**Figure 4:**
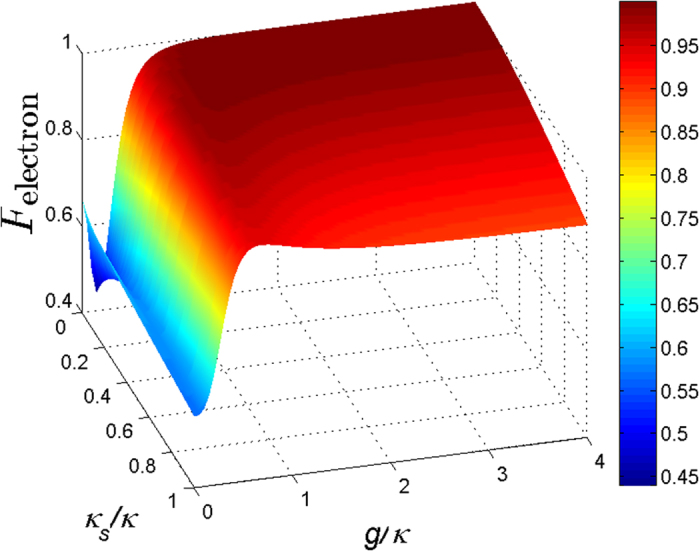
The fidelity of the electronic *W*-state fusion scheme versus the normalized coupling strengths *g*/*κ* and side leakage rate *κ*_*s*_*/κ*, where we have set *γ* = 0.1*κ*.

**Figure 5:**
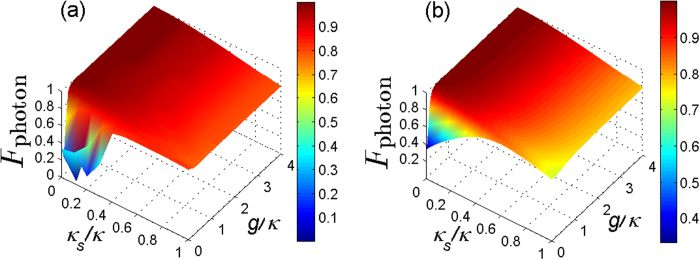
The fidelity of the photonic *W*-state fusion scheme versus the normalized coupling strengths *g*/*κ* and side leakage rate *κ*_*s*_*/κ*. (**a**) The fidelity corresponding to that the measurement result of the electron spin is 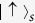. (**b**) The fidelity corresponding to that the measurement result of the electron spin is 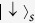. Here we have set *γ* = 0.1*κ*.

The fidelity of the fusion scheme is 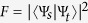, in which 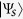 and 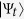 represent the final states of the present fusion scheme in the realistic condition and the ideal condition, respectively. Figures 4 and 5 are the fidelities of electronic and photonic *W*-state fusion schemes (*F*_electron_ and *F*_photonic_), respectively, which show our schemes can be achieved with high fidelities. Nevertheless, the cavity side leakage and cavity field decay have obvious impact on the fusion-scheme fidelities. Fortunately, the strong coupling of the QD-microcavity system has been observed in^42^,^43^,^44^,^45^. And the improvement of fabrication techniques can suppress the side leakage which reported 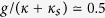 and 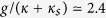. In our scheme, if setting *κ*_*s*_ = 0.5*κ*, *g* = 2.5*κ*, (i.e. 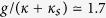 which is the strong coupling regime) we can obtain *F*_electron_ = 99.99%, 
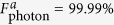
, and 
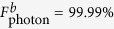
; even when setting *κ*_*s*_ = 1.0*κ*, *g* = 0.4*κ*, (i.e. *g*/(*κ* + *κ*_*s*_) = 0.2 which is the weak coupling regime) we also can obtain *F*_electron_ = 87.55%, 
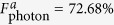
, and 
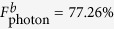
. Therefore, our scheme can work well in both the weak coupling and the strong coupling regimes.

In addition, the electron spin decoherence and the exciton dephasing could also effect the fidelity. Exciton dephasing reduces the fidelity by the amount of 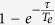, where 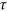 is the photon life time in the cavity and *T*_*e*_ is the exciton coherence time^26^,^27^. The optical dephasing reduces the fidelity only a few percent that is because in a self-assembled In(Ga)As-based QD the time scale of the excitons can reach hundreds of picoseconds^45^,^46^,^47^. The effect of the spin dephasing is mainly due to the hole-spin dephasing, while the hole spin coherence time is at least three orders of magnitude longer than the cavity photon lifetime^48^, so the spin dephasing can be safely neglected.

Obviously, in the whole fusion process, the fused large *W* state is from the items of the initial state with the electron (photon) 1 and 2 in the states 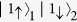, 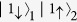, and 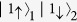 (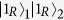, 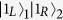, and 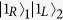). While the item with the electron (photon) 1 and 2 in 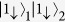 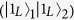 would become two smaller *W* states after measuring the photon 1 (electron 1), which can be recycled using the same fusion mechanism. Therefore the success probability *P*_*s*_ and the recyclable probability *P*_*r*_ are written as


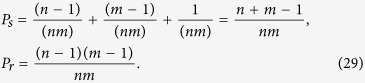


Therefore, the fusion schemes for electronic and photonic *W* state could be effectively implemented with the success probability (*n* + *m* − 1)/*nm*.

## Methods

### Input-output relation of QD double-sided cavity system

The reflection and transmission coefficients of the QD-cavity system can be calculated from the Heisenberg equations of motion for the cavity field operator 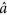 and *X*^*−*^ dipole operator *σ*_−_ in the interaction picture^49^


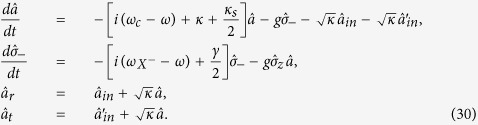


where 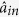, 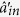 and 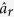, 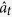 are the two input and the two output fields operators of the double-side cavity, respectively. And other parameters are the same as Eq. (25). The reflection and transmission coefficients in Eq. (25) can be obtained in the approximation of weak excitation where the charged QD is predominantly in the ground state with 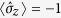.

### Manipulation and measurement of the electron spin in QD

The Hadamard transformation and projective measurement on the electron spin are needed in the present schemes. The Hadamard transformation {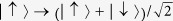, 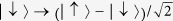} can be implemented by using a *π*/2 microwave or optical pulse^23^,^50^,^51^. The projection measurement of the electron spin can be achieved with the help of an auxiliary circularly polarized photon. If a right-circularly polarized photon 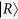 is initially sent into the cavity along the *z* axis, after interacting with QD-cavity system, the joint state of the photon and electron becomes


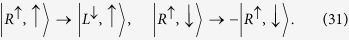


Obviously, the projection measurement of the electron spin can be completed by detecting the reflection and transmission of the photon. The electron spin is projected into the state 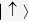 for photon’s reflection; the electron spin is projected into the state 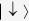 for photon’s transmission.

## Additional Information

**How to cite this article**: Han, X. *et al.* Effective *W*-state fusion strategies for electronic and photonic qubits via the quantum-dot-microcavity coupled system. *Sci. Rep.* **5**, 12790; doi: 10.1038/srep12790 (2015).
